# Maternal Health Outcomes of Digital Health Interventions in Low- and Middle-Income Countries in Southeast Asia: Scoping Review

**DOI:** 10.2196/92476

**Published:** 2026-11-03

**Authors:** Eun K Yang, Moonju Lee, Helen Park-Chong, Saisuree Namluck, Sun S Kim

**Affiliations:** 1 Patricia A. Chin School of Nursing, California State University, Los Angeles, 5151 State University Drive, Los Angeles, CA, 90032, United States; 2 Marcella Niehoff School of Nursing, Loyola University Chicago, Chicago, IL, United States; 3 NewYork-Presbyterian/Columbia University Medical Center, New York, NY, United States; 4 Columbia University School of Nursing, New York, NY, United States; 5 Donna and Robert Manning College of Nursing and Health Sciences, University of Massachusetts Boston, Boston, MA, United States

**Keywords:** maternal health outcomes, digital health interventions, low- and middle-income countries, Southeast Asia, scoping review

## Abstract

**Background:**

Digital health interventions (DHIs) have gained increasing attention as strategies to improve maternal health in low- and middle-income countries (LMICs). Previous reviews of DHIs have included studies from Southeast Asia (SEA) but have not examined maternal health outcomes in SEA LMICs as a distinct regional evidence base.

**Objective:**

This scoping review aimed to map maternal health outcomes of DHIs in SEA LMICs using an adapted proximal-intermediate-distal (PID) framework, describe DHI characteristics, and summarize contextual factors related to target groups and intervention settings.

**Methods:**

The review followed the PRISMA-ScR (Preferred Reporting Items for Systematic Reviews and Meta-Analyses Extension for Scoping Reviews) and the Joanna Briggs Institute guidance. PubMed, CINAHL, and Scopus were searched for English-language interventional studies evaluating DHIs that were published from January 2005 through May 2025. Google Scholar, reference lists, and the Index Medicus for the South-East Asia Region were searched as supplementary sources. Maternal health outcomes were mapped using an adapted PID framework, and all results were summarized descriptively.

**Results:**

Twenty-five studies from 7 SEA LMICs were included. Mobile apps and SMS were the most commonly used technologies, reported in 22 (88%) studies. Fourteen (56%) studies reported maternal health outcomes across more than one PID domain. Intermediate outcomes were reported most frequently (n=23, 92%), followed by proximal knowledge outcomes (n=10, 40%) and distal biomedical and clinical outcomes (n=8, 32%). Findings related to proximal knowledge outcomes were predominantly significant, whereas intermediate and distal outcomes showed mixed results. All but one study (n=24, 96%) recruited participants through existing health care services, and most interventions were implemented in primary care settings (n=17, 68%).

**Conclusions:**

Maternal health outcomes were represented across all 3 PID domains, with intermediate outcomes reported most frequently and distal biomedical and clinical outcomes least frequently. Future studies should give more attention to distal biomedical and clinical outcomes, as well as underrepresented SEA LMICs.

## Introduction

Maternal mortality remains a major indicator of health system equity and a persistent global public health concern. Although the number of maternal deaths has declined over recent decades, approximately 287,000 maternal deaths still occur each year from complications related to pregnancy and childbirth [1]. In 2020, the global maternal mortality ratio (MMR) was estimated at 223 deaths per 100,000 live births, with nearly 90% of these deaths occurring in low- and middle-income countries (LMICs) [1]. Likewise, maternal mortality remains a considerable burden in Southeast Asia (SEA) [1]. The region comprises 11 countries, including Brunei Darussalam, Cambodia, Indonesia, the Lao People’s Democratic Republic (Lao PDR), Malaysia, Myanmar, the Philippines, Singapore, Thailand, Timor-Leste, and Vietnam. Of these, 9 countries, excluding Brunei Darussalam and Singapore, are classified as LMICs [2]. MMRs vary widely across LMICs in SEA. For instance, estimates from 2020 indicate that Malaysia and Thailand reported MMRs of 21 and 29 per 100,000 live births, respectively, which are below the global average but higher than the estimated MMR of 11 per 100,000 live births in high-income countries [1]. In contrast, Timor-Leste and Cambodia reported considerably higher ratios of 204 and 218 per 100,000 live births, respectively [1]. These disparities may reflect differences in health care system structures and contextual factors that shape access to care and health care–seeking behaviors [3].

Digital health interventions (DHIs) have gained visibility as potentially effective and scalable strategies to improve maternal health, particularly in resource-limited settings such as LMICs [4]. DHIs encompass a broad range of technologies, including mobile messaging services, smartphone apps, telemedicine platforms, wearable devices, and digital referral or decision-support systems [4]. In maternal health care delivery, these technologies have been used to support appointment reminders, health education, communication with providers, and facilitation of service use, including antenatal care visits and facility-based delivery [3,5].

The COVID-19 pandemic accelerated the digitalization of health systems worldwide, as governments sought to maintain continuity of care amid service disruptions [6]. In SEA, this shift also created new opportunities to improve digital care delivery [6,7]. DHIs may strengthen existing health services [4] while also improving access in rural and resource-constrained settings [3,5]. Regional initiatives, including the Association of Southeast Asian Nations (ASEAN) Health Cluster on Strengthening Health Systems and the World Health Organization (WHO) Global Strategy on Digital Health, have also provided additional momentum for digital transformation in SEA [6,7].

Previous reviews have examined digital maternal health interventions across multiple LMIC regions [3,8]. Although these reviews included studies from SEA LMICs, they did not examine the region as a distinct evidence base or synthesize maternal health outcomes within this context. To our knowledge, no previous scoping review has specifically addressed maternal health outcomes of DHIs in SEA LMICs.

WHO guidance provides a useful conceptual basis for organizing maternal health outcomes according to their relationship to an intervention and its longer-term aims. The guidance distinguishes short-term or proximal outcomes as changes occurring at or soon after an intervention, intermediate outcomes as subsequent changes that may include behavioral responses, and distal or long-term outcomes as broader changes related to the ultimate aims of the intervention [9]. Drawing on these distinctions, this review adapted and operationalized proximal, intermediate, and distal (PID) outcome categories as an organizing framework for consistently mapping maternal health outcomes. Proximal outcomes were operationalized as maternal health knowledge; intermediate outcomes included psychosocial outcomes, attitudinal outcomes, perceived capability, behavioral outcomes, and service utilization; and distal outcomes included biomedical and clinical measures of maternal health status, such as findings from clinical examinations, diagnostic tests, and laboratory measures.

This scoping review aimed to map reported maternal health outcomes using the adapted PID framework, describe DHI characteristics, and summarize contextual factors. Maternal health outcomes were examined by their distribution within the adapted PID framework and the direction and statistical significance of reported findings. DHI characteristics included intervention modality, contact frequency, intervention duration, and follow-up timing. Contextual factors included target groups and intervention settings.

## Methods

This review was conducted following the Preferred Reporting Items for Systematic Reviews and Meta-Analyses Extension for Scoping Reviews (PRISMA-ScR) and the Joanna Briggs Institute (JBI) methodological guidance (Checklist 1) [10,11]. The review protocol was registered with the Open Science Framework before title and abstract screening began [12].

### Study Design

A scoping review methodology was used to systematically map the evidence from the included studies. The review process involved formulating the review question, identifying relevant studies, selecting eligible studies, charting the data, collating the results, and summarizing the findings.

### Eligibility Criteria

Eligibility criteria were established based on the JBI population-concept-context (PCC) framework [11]. Studies were included if all PCC elements were met, along with additional criteria for outcomes, study characteristics, language, and publication period. The review focused on English-language studies published between January 2005 and May 2025 to encompass the period of widespread adoption of mobile- and internet-based technologies in SEA. Table 1 summarizes the full criteria.

**Table 1.** Population-concept-context (PCC) framework and eligibility criteria.

PCC element	Inclusion criteria	Exclusion criteria
Population	Individuals who were pregnant, were up to 12 months post partum, and had experienced pregnancy loss or abortion.	Newborns, infants, and individuals who are not pregnant, were not within the postpartum period, and had not experienced pregnancy loss or abortion.
Concept	Digital health interventions, including mobile phones, SMS, mobile or web apps, telemedicine, and wearable devices [13].	Interventions with no digital health component.
Context	Southeast Asian low- or middle-income countries [2]: Cambodia, Indonesia, Lao PDR^a^, Malaysia, Myanmar, the Philippines, Thailand, Timor-Leste, and Vietnam. Country eligibility was determined using the World Bank Income classifications current at the time the literature search was conducted.	Countries outside Southeast Asia or countries classified as high-income countries [2].
Outcomes	At least one maternal health outcome mapped to the adapted PID^b^ framework, including knowledge, psychosocial, attitudinal, perceived capability, behavioral, service utilization, or biomedical outcomes.	DHI^c^ outcomes without individual-level maternal health outcomes.
Study characteristics	Interventional studies evaluating DHIs.	Studies without a DHI.
Language and time frame	English-language full texts published between January 2005 and May 2025.	Non-English publications and studies published outside of the time frame.

a Lao PDR: Lao People’s Democratic Republic.

b PID: proximal, intermediate, and distal.

c DHI: digital health intervention.

### Search Strategy

An academic librarian collaborated with the review team to develop and iteratively refine the search strategy. The search combined controlled vocabulary and free-text keywords related to maternal health, DHIs, and SEA. PubMed, CINAHL, and Scopus were searched as the primary databases. A supplementary search of Google Scholar and the WHO Global Index Medicus, using the Index Medicus for the South-East Asia Region (IMSEAR), was conducted to expand the coverage of regional and gray literature. The reference lists of relevant review articles were also screened for additional studies. Full search strings, filters, dates, and source-specific procedures are provided in Multimedia Appendix 1.

### Study Selection

All records retrieved through database and supplementary searches were imported into Covidence (Veritas Health Innovation) for management and duplicate removal. The searches retrieved 1133 records from PubMed (n=455), Scopus (n=300), CINAHL (n=110), Google Scholar (n=200), and IMSEAR (n=68). Of these, 234 duplicate records were removed, leaving 899 records for title and abstract screening.

The primary reviewer (EKY) screened all titles and abstracts for eligibility using Covidence, and each record was independently screened by either ML or HP-C as the second reviewer. During the full-text screening, each article was independently assessed by at least 2 of the 3 reviewers (EKY, ML, and HP-C). Prior to screening, the reviewers completed a calibration exercise using 20 abstracts to practice applying the eligibility criteria. Interrater agreement was assessed using Cohen κ, as generated by Covidence. At the title and abstract screening stage, pairwise agreement was κ=0.35 (n=116) for EKY and ML and κ=0.69 (n=783) for EKY and HP-C. At the full-text screening stage, pairwise agreement was κ=0.66 (n=70) between EKY and ML, κ=0.70 (n=72) between EKY and HP-C, and κ=0.75 (n=17) between ML and HP-C [14]. Disagreements between reviewers were initially resolved through discussion among the 3 reviewers (EKY, ML, and HP-C). When consensus could not be reached, the senior author (SSK) was consulted to make the final inclusion decision.

Full-text screening identified 25 studies that met the inclusion criteria. The primary reasons for exclusion were non–SEA LMIC settings (n=37), wrong populations (n=36), the absence of DHI components (n=28), wrong outcomes (n=11), and wrong study designs (n=6). One study was excluded because its full text could not be retrieved despite several attempts.

### Data Extraction

#### Data Charting Process

Data from all 25 included studies were independently charted by at least 2 of the 3 reviewers (EKY, ML, and HP-C) using a data-charting template in Covidence. The data-charting template was pilot-tested with 5 studies and refined iteratively before and during charting. After verification, terminology and categorical codes were standardized across studies, including the classification of maternal health outcomes within the adapted PID framework.

#### Data Items

The charted study characteristics included the author and year, country, study design, participant population, and sample size. Data relevant to the review objectives were organized into 3 areas: DHI characteristics, maternal health outcomes, and contextual factors.

DHI characteristics included intervention modality, contact frequency, intervention duration, and follow-up timing. Intervention modality referred to the type of technology used, such as SMS, mobile apps, augmented reality, or interventions combining multiple digital modalities. The components delivered to the intervention group and, when applicable, the conditions received by the comparator group were also charted. Contact frequency described how often the intervention was delivered; continuous availability for participant-initiated use was charted as a separate category. Intervention duration described the length of time during which the intervention was provided. Follow-up timing referred to the time points at which outcome data were collected.

Maternal health outcomes were organized using the adapted PID framework. Each outcome was coded as proximal, intermediate, or distal based on the operational definition of the measured maternal health construct. Knowledge was coded as proximal, representing maternal understanding or awareness of health-related information. Intermediate outcomes reflected maternal experiences, perceptions, behaviors, and use of health services and were classified into 5 groups: psychosocial outcomes, attitudinal outcomes, perceived capability, behavioral outcomes, and service utilization. Distal outcomes included biomedical and clinical measures reflecting maternal health status, such as physiological, hematologic, metabolic, anthropometric, diagnostic, morbidity, and mortality indicators. For each outcome, the direction and statistical significance of the finding were charted.

Contextual factors included the target population and setting of a DHI. The target population was categorized as (1) an existing patient group who had been using the health care service where the DHI was implemented, (2) an underserved group who could not access the existing health care service, (3) both groups, or (4) not reported. The intervention setting was the health care setting in which the DHI was implemented.

### Data Synthesis

A narrative synthesis was performed using descriptive and categorical methods. First, the study and DHI characteristics were described. Second, maternal health outcomes were mapped using the adapted PID framework. Lastly, the target populations and intervention settings in which the DHIs were implemented were summarized. The synthesis revealed patterns and identified evidence gaps. No meta-analysis was conducted.

### Quality Appraisal and Ethical Considerations

A quality appraisal or risk-of-bias assessment was not conducted, which is consistent with the purpose and methodology of a scoping review [10,15]. This review used publicly available literature and did not involve human participants or primary data collection; therefore, ethics approval was not required.

## Results

### Characteristics of the Studies

Figure 1 presents the full study-selection process. Table 2 summarizes the characteristics of the included studies, DHI modality, contact frequency, intervention duration, follow-up timing, and reported maternal health outcomes. The 25 included studies were published between 2008 and 2025, with 18 (72%) published from 2020 onward [16-33]. Figure 2 shows the distribution of included studies by publication year.

**Figure 1.**
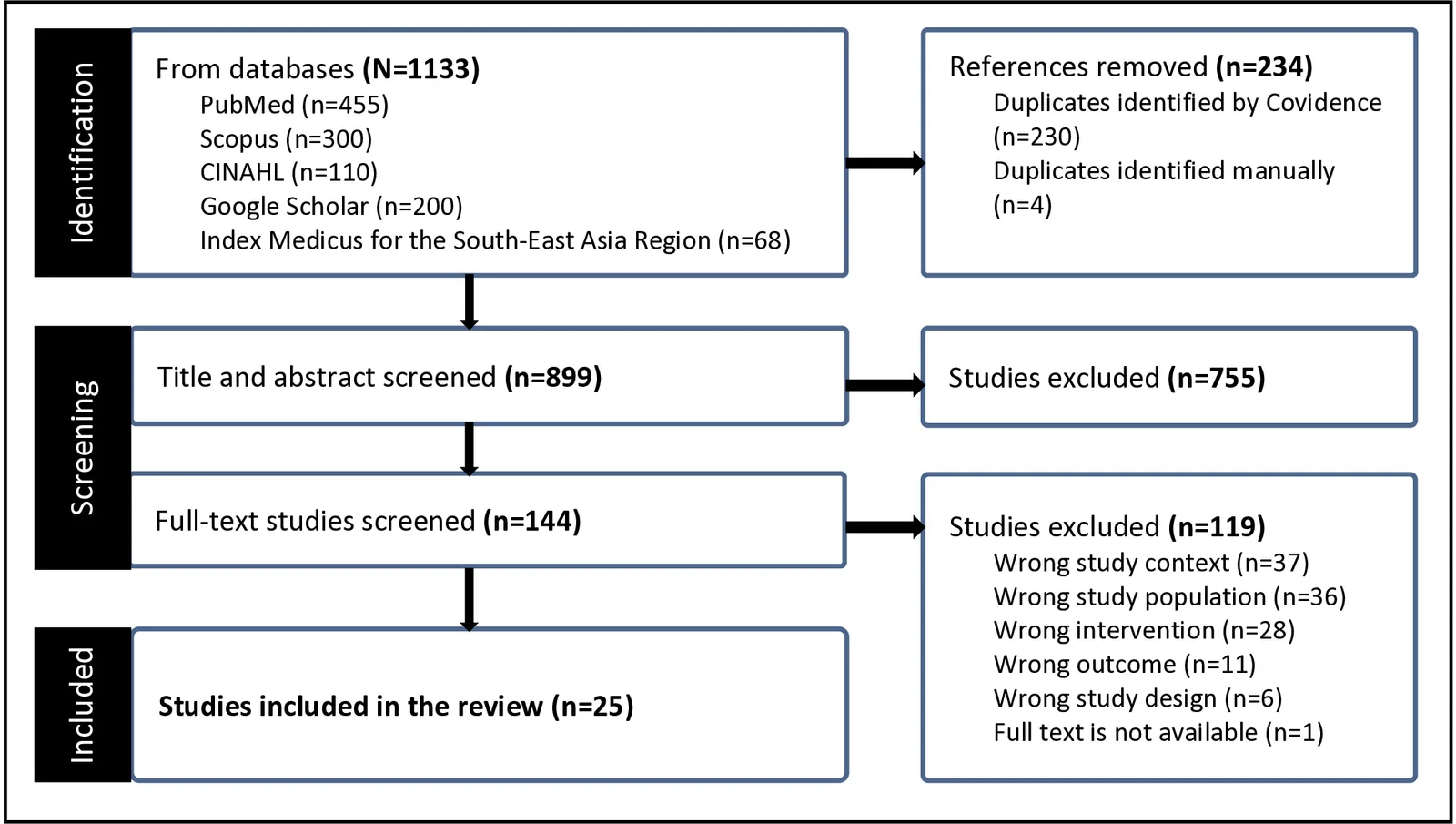
PRISMA-ScR (Preferred Reporting Items for Systematic Reviews and Meta-Analyses Extension for Scoping Reviews) flow diagram for study selection.

**Table 2.** Characteristics of included studies, digital health interventions, and reported maternal health outcomes.

Author, year	Country	Study design	Study participants (n)	Intervention group vs control group	Contact frequency and duration; follow-up	Results: proximal outcome (P), intermediate outcome (I), and distal outcome (D)^a^
Anitasari and Andrajati [34], 2017	Indonesia	Quasi-experimental	Pregnant women (74)	SMS reminders vs leaflets	Weekly for a month; postintervention at 1 month	P: —^b^ I: No difference in iron supplement adherence D: No difference in hemoglobin levels
Anwar et al [16], 2024	Indonesia	Double-blind RCT^c^	Pregnant women with anemia (68)	SMS education plus iron supplementation vs iron supplementation alone	Weekly for 4 weeks; postintervention at day 29	P: Knowledge increased pre-post in SMS group (*P*=.047); no difference in iron supplementation knowledge between groups I: No difference in iron supplement consumption between groups D: —
Arifah et al [17], 2023	Indonesia	Single-blind RCT	Pregnant women (44)	SMS education and reminders and usual care^d^ vs usual care	Daily for 7 weeks; postintervention	P: Higher anemia prevention knowledge in the intervention group than in the control group (*P*=.007) I: Higher iron intake in the intervention group than in the control group (*P*=.003); no difference in anemia prevention diet consumption between the groups D: —
Gerdts et al [18], 2020	Indonesia	No blind RCT	Pregnant women (242)	Mobile app plus usual care vs usual care	24-hour access^e^ for 24 days; postintervention	P: — I: No difference in feelings of preparedness, confidence, and support for medication abortion between the 2 groups (*P* value: N/A^f^) D: —
Haslin et al [19], 2021	Indonesia	Quasi-experimental	Pregnant women (52)	Mobile app vs handbook	24-hour access for 2 weeks; postintervention	P: Higher pregnancy danger sign knowledge in the intervention group than in the control group (*P*<.001) I: — D: —
Latifah et al [20], 2024	Indonesia	No blind RCT	Pregnant and postpartum women (128)	Mobile app plus Quran recitation vs no treatment	Daily until 2 months post partum; postintervention	P: — I: Lower postpartum depression scores in the intervention group than in the control group (*P* value: N/A) D: —
Manurung et al [21], 2022	Indonesia	Quasi-experimental	Pregnant women (64)	Mobile app vs illustrated educational module	24-hour access for 3 months; postintervention	P: Higher pregnancy-related knowledge in the intervention group than in the control group (*P*<.001) I: More positive attitudes (*P*=.003) and better practices in the intervention group than in the control group (*P*<.001) D: —
Mulyani et al [22], 2023	Indonesia	Quasi-experimental	Pregnant women (86)	AR^g^ media (single group)	Weekly for 5 months; postintervention	P: Increased healthy lifestyle knowledge after the intervention (*P* value threshold: <.05) I: Increased healthy lifestyle behavior (*P* value threshold: <.05) and no significant change in healthy lifestyle attitudes after the intervention D:
Nurcahyani et al [23], 2023	Indonesia	Quasi-experimental	Pregnant women (110)	Mobile app plus usual care vs Flipcharts plus usual care	24-hour access until 42 days post partum; postintervention	P: — I: The odds of postpartum contraceptive use were 2.4 (95% CI 1.08‐5.43; *P* value threshold: <.05) times as high in the intervention group as in the control group D: —
Petralina et al [24], 2025	Indonesia	Quasi-experimental	Pregnant women (350)	Mobile app plus usual care vs usual care	—; postintervention	P: — I: Greater improvements in self-efficacy, future outlook, self-esteem, support and assurance, and decision-making in the intervention group than in the control group (all *P*=.001); no difference in mobilization D: —
Rukmaini and Masrul [35], 2018	Indonesia	Quasi-experimental	Pregnant women (86)	Mobile app (single group)	Daily for 30 days; postintervention	P: — I: Increased adherence to iron tablet intake after the intervention (*P*=.002) D: —
Suarayasa et al [25], 2021	Indonesia	Quasi-experimental	Pregnant women (86)	Mobile app vs no intervention	—; postintervention	P: Higher knowledge of pregnancy risk detection in the intervention group than in the control group (*P*<.001) I: More favorable attitudes, action, and skills in the intervention group than in the control group (all *P*<.001) D: —
Susilawati et al [26], 2021	Indonesia	Quasi-experimental	Pregnant women (50)	Mobile app (single group)	Daily for 30 days; postintervention	P: — I: Adherence to daily iron supplementation was 78% (*P* value: N/A). D: Mean hemoglobin levels increased from 9.64 (SD 0.928) to 10.38 (SD 0.920) g/dL after the intervention (*P*=.01)
Trias et al [27], 2024	Indonesia	Quasi-experimental	Pregnant women (60)	Mobile app vs fetal movement card	Daily for 7 days; postintervention	P: — I: Anxiety decreased significantly after both the mobile app and the fetal movement card intervention (both *P*<.001); no difference in fetal movement monitoring adherence between the groups D: —
Triastin et al [28], 2021	Indonesia	Quasi-experimental	Pregnant women (54)	Mobile app vs usual care	Daily for 2 weeks; postintervention	P: Higher childbirth preparation knowledge in the intervention group than in the control group (*P*<.001) I: — D: —
Choakprasanchai et al [29], 2024	Thailand	Quasi-experimental	Pregnant women (70)	Mobile app plus usual care vs usual care	3 sessions over 4 weeks; 2 and 4 weeks postintervention	P: — I: Higher self-management behavior in the intervention group at 4 weeks (*P* value threshold: <.05); no difference in quality of life D: Increased hemoglobin and hematocrit levels, greater gestational weight gain, and fewer UTIs^h^ in the intervention group than in the control group (*P* value threshold: <.05)
Jareethum et al [36], 2008	Thailand	Single-blind RCT	Pregnant women (68)	SMS plus usual care vs usual care	Twice a week from 28 weeks’ gestation until delivery; immediate post partum	P: — I: Higher satisfaction during antenatal (*P*<.001) and perinatal periods (*P*=.007), higher confidence (*P*=.001), and lower anxiety (*P*=.002) during the antenatal period; no significant differences in anxiety and confidence during the perinatal period D: No differences in gestational age at delivery, preterm delivery, or mode of delivery between the 2 groups
Nooied et al [30], 2023	Thailand	Single-blind RCT	Pregnant women (128)	Mobile app plus 2 nurse-led sessions over 2 weeks vs usual care	24-hour access for 2 weeks; postintervention	P: — I: Higher childbirth self-efficacy and lower fear of childbirth in the intervention group than in the control group (all *P*<.001) D: —
Xuto et al [31], 2022	Thailand	Single-blind RCT	Pregnant women (66)	SMS plus usual care vs usual care	56 messages delivered between 13 and 40 gestational weeks; after giving birth	P: — I: Higher physical activity behavior scores (*P*=.01) and lower anxiety (*P*=.02) in the intervention group than in the control group D: No differences in gestational weight gain, hemoglobin levels, glucose challenge test results, pregnancy complications, gestational age at birth, or mode of delivery between the 2 groups
Navarro et al [37], 2019	Philippines	Quasi-experimental	Pregnant women (88)	SMS (single group)	Seminar, twice a week messages for 8 weeks; postintervention	P: Increased UTI-related knowledge after the intervention (*P*<.001) I: No difference in personal hygiene practices but increased water intake (*P*<.001) D: Decreased UTIs (*P*<.001)
Sarmiento et al [38], 2019	Philippines	Single-blind RCT	Pregnant women with gestational diabetes mellitus (308)	SMS vs usual care	Twice a week at 4, 8, and 10 weeks post partum; 12 weeks post partum	P: — I: No difference in postpartum follow-up between the groups at 12 weeks D: —
Smith et al [39], 2015	Cambodia	Single-blind RCT	Postabortion women (500)	Automated interactive voice messages and phone support plus usual care vs usual care	Every 2 weeks for 3 months; at 4 and 12 months post abortion	P: — I: Higher effective contraceptive use at 4 months (64% vs 46%; RR^i^=1.39, 95% CI 1.17‐1.66), but no difference at 12 months (50% vs 43%; RR=1.16, 95% CI 0.92‐1.47); higher long-acting contraceptive method at 4 months (RR=3.35, 95% CI 2.07‐5.40) and at 12 months (RR=2.08, 95% CI 1.27‐3.42) in the intervention group D: No differences in repeat pregnancy and repeat abortion at 4 or 12 months between the 2 groups
Jaffar et al [32], 2022	Malaysia	Single-blind RCT	Pregnant women with urinary incontinence (26)	Mobile app vs usual care	Three times daily for 8 weeks; at 1- and 2-month follow-up postintervention	P: Higher PFMT^j^ knowledge in the intervention group than in the control group at 1 month (*P*=.01), but not at 2 months I: Higher self-efficacy at 1 month (*P*=.04) and more positive attitudes at 2 months (*P*=.03) in the intervention group than in the control group, but no difference in PFMT practice or adherence between the 2 groups D: No difference in urinary incontinence symptom severity at 1 month or 2 months after intervention
Thompson et al [40], 2019	Timor-Leste	Quasi-experimental	Postpartum women (581)	SMS and 2-way communication vs no mobile health program in the control district	Twice a week until 6 months post partum; within 18 months postintervention	P: — I: Higher odds of skilled birth attendance (OR^k^=1.9, 95% CI 1.1-3.2; *P*=.02), facility delivery (OR=1.9, 95% CI 1.1-3.6; *P*=.02), and postpartum care within 2 days of delivery (OR=4.7, 95% CI 2.4-9.0; *P*=.001); no difference in receiving 4 or more antenatal care visits D: —
Dao et al [33], 2023	Vietnam	Cluster RCT	Pregnant women (832)	SMS vs usual care	2‐3 times a week during weeks 5‐42 of pregnancy; postintervention	P: Higher knowledge of pregnancy danger signs and antenatal supplements in the intervention group than in the control group (convulsions: *P*=.05; iron awareness: *P*=.01) I: Higher folic acid, calcium, and DHA^l^ intake (*P* value threshold: <.01), but no difference in iron intake; significant between-group difference over time in monthly weight checks, blood tests, and urine tests (*P* value threshold: <.01), but not monthly blood pressure checks; higher self-rated health in the intervention group (*P* value: N/A) D: —

a Exact *P* values are reported where available. Values below .001 are reported as *P*<.001. Where studies reported thresholds only, this is indicated by “*P* value threshold.” Numerical *P* values have been omitted for nonsignificant findings.

b Not available.

c RCT: randomized controlled trial.

d “Usual care” refers to care described in the original studies as routine, standard, or usual care; its content was not consistently defined and may have varied across studies and settings.

e “24-Hour access” indicates continuous availability for participant-initiated use without a prescribed frequency and does not imply continuous or daily use.

f N/A indicates that an exact numerical value or threshold is not reported.

g AR: augmented reality.

h UTIs: urinary tract infections.

i RR: relative risk.

j PFMT: pelvic floor muscle training.

k OR: odds ratio.

l DHA: docosahexaenoic acid.

**Figure 2.**
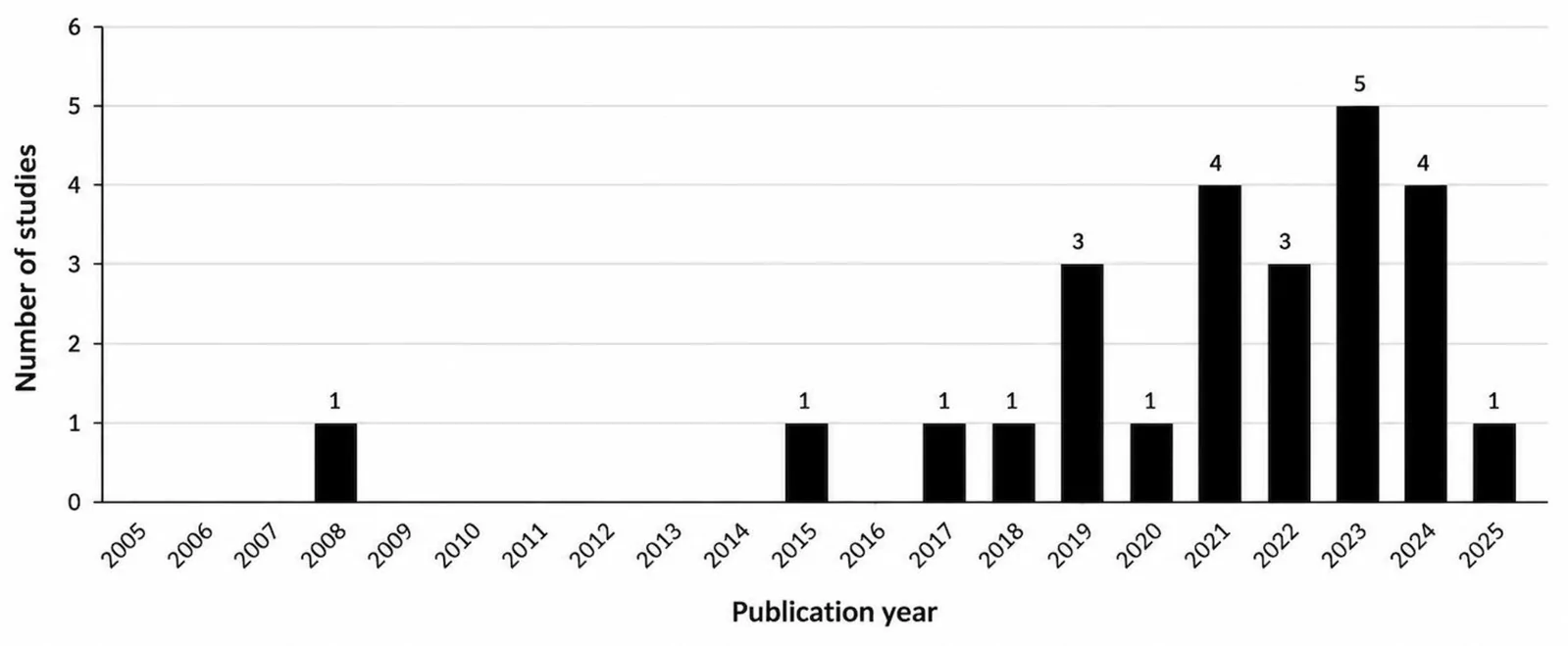
Number of included studies by publication year. The 2025 count includes studies published up to May 2025.

Studies were conducted in 7 SEA LMICs. Indonesia contributed the largest number of studies (n=15, 60%) [16-28,34,35], followed by Thailand (n=4, 16%) [29-31,36], the Philippines (n=2, 8%) [37,38], and Cambodia [39], Malaysia [32], Timor-Leste [40], and Vietnam [33] (n=1, 4% each). No eligible studies were identified from Lao PDR or Myanmar. Fourteen (56%) studies used quasi-experimental designs [19,21-29,34,35,37,40], and 11 (44%) used randomized controlled trial designs [16-18,20,30-33,36,38,39]. Sample sizes were most often 100 or fewer (n=16, 64%) [16,17,19,21,22,25-29,31,32,34-37], followed by 101 to 500 (n=7, 28%) [18,20,23,24,30,38,39] and more than 500 participants (n=2, 8%) [33,40]. Twenty-two (88%) studies enrolled only pregnant participants [16-19,21-38]. The remaining studies included pregnant and postpartum participants [20], postabortion participants [39], and postpartum participants [40] (n=1, 4% each).

### Characteristics of DHIs

Mobile apps (n=14, 56%) were the most common DHI modality [18-21,23-30,32,35], followed by SMS (n=8, 32%) [16,17,31,33,34,36-38]. The remaining interventions used augmented reality [22], automated interactive voice messaging with phone support [39], or a combination of SMS and voice communication (n=1, 4% each) [40]. Contact frequency varied across studies. Daily contact was reported in 7 (28%) studies [17,20,26-28,32,35], multiple contacts per week were reported in 5 (20%) studies [33,36-38,40], and weekly contact was reported in 3 (12%) studies [16,22,34]. Three (12%) studies used other scheduled frequencies: 3 sessions over 4 weeks [29], 56 messages between 13 and 40 gestational weeks [31], and contact every 2 weeks for 3 months [39]. Five (20%) studies provided 24-hour access without a prescribed frequency [18,19,21,23,30], and 2 (8%) did not report contact frequency [24,25]. Intervention duration also varied among studies. Ten (40%) interventions lasted 1 month or less [16,18,19,26-30,34,35], while 6 (24%) studies lasted more than 1 month and up to 5 months [17,21,22,32,37,39]. Seven (28%) studies defined intervention duration by gestational or postpartum timing rather than a fixed period [20,23,31,33,36,38,40], and 2 (8%) studies did not report duration [24,25].

### Maternal Health Outcomes

#### Overview

Maternal health outcomes were categorized into PID domains using the adapted PID framework. Fourteen (56%) studies reported maternal health outcomes across more than one PID domain [16,17,21,22,25,26,29,31-34,36,37,39]. Outcomes were summarized descriptively, including the direction and statistical significance of the findings reported in the original studies.

#### Proximal Outcomes

Maternal knowledge outcomes were reported in 10 of 25 (40%) studies [16,17,19,21,22,25,28,32,33,37]. Knowledge addressed anemia prevention and iron supplementation [16,17]; the importance of antenatal supplements, including folic acid, iron, calcium, and docosahexaenoic acid [33]; pregnancy-related health and care [21]; healthy lifestyles [22]; pregnancy danger signs and risk detection [19,25,33]; childbirth preparation [28]; urinary tract infection prevention [37]; and pelvic floor muscle training [32]. Eight studies reported significantly higher knowledge in the intervention group than in the control group or a significant increase in knowledge following the intervention relative to baseline [17,19,21,22,25,28,33,37]. Findings in the remaining 2 studies varied by comparison or follow-up time point. Knowledge of iron supplementation increased significantly from baseline in the intervention group; however, postintervention knowledge did not differ between the intervention and control groups [16]. Knowledge of pelvic floor muscle training was higher in the intervention group at the 1-month follow-up compared with the control group, but the between-group difference was no longer significant at the 2-month follow-up [32].

#### Intermediate Outcomes

Intermediate outcomes were reported in 23 of 25 (92%) studies [16-18,20-27,29-40]. Outcomes were grouped into 5 categories: (1) psychosocial outcomes, (2) attitudinal outcomes, (3) perceived capability, (4) behavioral outcomes, and (5) service utilization. Twelve (48%) studies reported outcomes in at least 2 of the 5 intermediate-outcome categories [18,21,22,24,25,27,29-33,36].

Psychosocial outcomes were reported in 9 (36%) studies [18,20,24,27,29-31,33,36]. Outcomes included perceived support [18,24], depression [20], self-esteem and assurance [24], anxiety [27,31,36], quality of life [29], satisfaction [36], fear of childbirth [30], and self-rated health [33]. Overall, DHIs were associated with improvements in depression scores [20], self-esteem, perceived support, and assurance [24], anxiety [27,31,36], satisfaction [36], fear of childbirth [30], and self-rated health [33]. However, findings varied by outcome variable, intervention modality, and contact frequency [18,24,27,29,30,33,36]. No significant improvements were found in perceived support related to medication abortion [18] or in quality of life [29] compared with usual care, and some improvements were limited to the antenatal period [36].

Attitudinal outcomes were reported in 4 (16%) studies [21,22,25,32]. Outcomes included attitudes related to pregnancy health and care [21], healthy lifestyles [22], early detection of pregnancy risk [25], and pelvic floor muscle training [32]. DHIs improved attitudes related to pregnancy health and care [21], early detection of pregnancy risk [25], and pelvic floor muscle training [32]. However, effects varied by outcome variable and follow-up time point, with no significant improvement in healthy lifestyle attitudes [22]; pelvic floor muscle training attitudes improved at 2-month follow-up but not at 1-month follow-up [32].

Perceived capability outcomes were reported in 5 (20%) studies [18,24,30,32,36]. Outcomes included preparedness [18], empowerment [24], self-efficacy [24,30,32], and confidence [18,36]. DHIs generally improved perceived capabilities related to early detection of pregnancy complications [24], antenatal care [36], childbirth [30], and pelvic floor muscle training [32]. However, effects varied by outcome variable and follow-up time point, with no significant changes in medication-abortion preparedness and confidence [18] or perinatal confidence [36], and some improvements in self-efficacy were not sustained over time [32].

Behavioral outcomes were reported in 16 (64%) studies [16,17,21-23,25-27,29,31-35,37,39]. Outcomes included iron supplementation adherence [16,17,26,33-35], healthy lifestyle practices [21,22,25], contraceptive use [23,39], fetal movement monitoring adherence [27], self-management behavior [29], physical activity [31], personal hygiene and water intake [37], pelvic floor muscle training practice and adherence [32], and other antenatal supplement intake [33]. DHIs generally improved maternal health practices [21], healthy lifestyle practices [22], contraceptive use [23,39], pregnancy risk detection [25], self-management [29], physical activity [31], water intake [37], and other antenatal supplement intake [33]. However, effects varied by target behavior and follow-up period, with no significant improvement in personal hygiene practices [37] and no significant between-group differences in fetal movement monitoring adherence [27] and pelvic floor muscle training practice and adherence [32]. Some contraceptive effects diminished over time [39]. Findings for iron supplementation were mixed across studies [16,17,26,33-35].

Service utilization outcomes were reported in 3 (12%) studies [33,38,40]. Outcomes included postpartum follow-up among women with gestational diabetes mellitus [38]; antenatal, delivery, and postpartum care [40]; and antenatal monitoring and diagnostic testing [33]. DHIs showed mixed effects on maternal health care utilization. SMS and 2-way communication increased skilled birth attendance, facility delivery, and timely postpartum care [40]. However, findings varied for postpartum follow-up [38], antenatal care [40], and routine monitoring [33].

#### Distal Outcomes

Distal outcomes were reported in 8 of 25 (32%) studies [26,29,31,32,34,36,37,39]. Outcomes included hematologic and glycemic measures [26,29,31,34], anthropometric measures [29,31], urinary outcomes [29,32,37], pregnancy and delivery outcomes [31,36], and reproductive outcomes [39]. Findings for hematologic, glycemic, and anthropometric outcomes varied across studies [26,29,31,34]. Mobile app interventions were associated with higher hemoglobin and hematocrit levels and greater gestational weight gain in some studies [26,29]. SMS interventions showed no significant effects on hemoglobin [31,34], glucose challenge results, or gestational weight gain [31] compared with usual care. DHIs were associated with reduced urinary tract infections in some studies [29,37], but no significant between-group differences were found in gestational age or mode of delivery [31,36], pregnancy complications [31], repeat pregnancy and abortion following postabortion care [39], or urinary incontinence severity [32].

### Contextual Factors

All but one (96%) study implemented a DHI targeting individuals already connected to their respective health care services. The exception [33] recruited participants from remote communities. Seventeen (68%) studies implemented DHIs in primary care settings [16,17,19-25,27,28,32-35,37,40]. Six (24%) studies were hospital-based [26,29-31,36,38]. One study was conducted in a community-based/nonclinical setting through a safe-abortion hotline [18], and another was conducted through outpatient reproductive health clinics [39].

## Discussion

### Principal Findings

This scoping review aimed to map maternal health outcomes reported in DHI studies conducted in SEA LMICs using the adapted PID framework. Outcomes were represented in all 3 domains, with intermediate outcomes reported most frequently (23/25, 92%), followed by proximal maternal knowledge outcomes (10/25, 40%) and distal biomedical and clinical outcomes (8/25, 32%). Most proximal knowledge outcomes showed significant improvement, while intermediate and distal outcomes showed mixed results. This pattern is consistent with other LMIC-focused reviews of DHIs in maternal health, which reported greater emphasis on knowledge, self-efficacy, and behavioral outcomes than on clinical outcomes [3,8].

Mobile apps and SMS were the predominant DHI modalities, accounting for 22 of 25 (88%) studies. Interventions varied in both contact frequency and duration. Contact frequency ranged from daily or multiple times per week to less frequent scheduled contact, while some interventions provided continuous access without a prescribed frequency. Intervention duration ranged from 1 month or less to several months, or periods defined by gestational or postpartum timing. These findings reflect diverse DHI design and delivery for maternal health in SEA LMICs. The prominence of mobile apps and SMS is consistent with a scoping review of 63 maternal and child health DHI studies in rural and underserved LMIC settings [3].

Intervention settings were identifiable for all studies. Most interventions were implemented in primary care settings, with fewer in hospitals, community-based nonclinical settings, or specialized outpatient reproductive health care settings. Only one study [33] recruited participants from remote communities without requiring an existing health care connection. Thus, this scoping review provides limited evidence on whether DHIs can expand access among populations not already connected to health care.

The studies were concentrated in a small number of SEA LMICs, with 60% (15/25) conducted in Indonesia and none were from Lao PDR or Myanmar. Indonesia, with a population of more than 280 million, is the world’s fourth-most-populous country and has a substantial potential user base for DHIs [41]. The country has established a national digital health transformation strategy that focuses on health data integration, digital health platforms, and a broader digital health ecosystem [42]. Its large population and expanding digital health environment may partly explain the concentration of DHI studies. Evidence from other SEA LMICs was limited, with only one study each from Cambodia, Malaysia, Timor-Leste, and Vietnam. This limited representation highlights gaps in the evidence base and constrains the generalizability of findings across the region.

### Implications for Practice

For maternal health nurses, midwives, and community health workers, this scoping review shows that DHIs have been integrated into primary care to support education, reminders, self-care, monitoring, follow-up, and continuity of care. While mobile app interventions showed improvements in some distal outcomes [26,29], DHIs should be tailored to the target population, care setting, and intended purpose [3,8]. To broaden the reach of DHIs beyond women who are already connected to health care services, maternal health professionals may consider community-based outreach and enrollment strategies.

DHI implementation should account for device access and connectivity, digital literacy, and privacy and communication needs [3,4]. Clear clinical pathways should specify how information is reviewed and when in-person assessment or referral is needed [4]. Digital approaches should complement, rather than replace, existing maternal health services, and appropriate nondigital options should remain available for individuals unable to participate in DHIs [4].

### Implications for Research

This scoping review highlights research priorities for maternal health DHIs in SEA LMICs. Attention to distal biomedical and clinical outcomes would broaden the evidence base beyond proximal and intermediate outcomes and provide a more complete understanding of outcomes within the PID framework. Research involving women with limited or no connection to existing health care services is also needed to determine whether DHIs can extend access to maternal health care beyond existing service users. Conducting studies in underrepresented SEA LMICs would strengthen the regional evidence base.

### Strengths and Limitations

This review provides a region-specific synthesis of maternal health outcomes reported in DHI studies conducted in SEA LMICs and systematically maps the outcomes using the adapted PID framework. Several limitations should be considered. The restriction to English-language literature may have excluded relevant studies. The review may also have missed unpublished or nonindexed literature. Several SEA LMICs were underrepresented in the evidence base, limiting regional representativeness. Variation in study designs, intervention characteristics, follow-up periods, and outcome measures limited comparison and synthesis across studies. Consistent with the scoping review design and objectives, no formal risk-of-bias or certainty-of-evidence assessment was conducted [10,11]. Accordingly, the findings describe the available evidence but do not establish its strength or certainty regarding intervention effectiveness.

### Conclusions

All 3 PID domains were represented among the maternal health outcomes reported in DHI studies conducted in SEA LMICs. Intermediate outcomes were reported most frequently, whereas distal biomedical and clinical outcomes were reported least frequently. Studies were concentrated in a limited number of countries. The regional evidence base could be strengthened by giving more attention to distal outcomes and underrepresented countries.

## Supplementary material

10.2196/92476 Multimedia Appendix 1 Full search strategies.

10.2196/92476 Checklist 1 PRISMA-ScR checklist.

## Abbreviations

ASEAN: Association of Southeast Asian Nations
DHI: digital health intervention
IMSEAR: Index Medicus for the South-East Asia Region
JBI: Joanna Briggs Institute
Lao PDR: Lao People’s Democratic Republic
LMIC: low- and middle-income country
MMR: maternal mortality ratio
PCC: population-concept-context
PID: proximal, intermediate, and distal
PRISMA-ScR: Preferred Reporting Items for Systematic Reviews and Meta-Analyses Extension for Scoping Reviews
SEA: Southeast Asia
WHO: World Health Organization
