# Supplementary material for: Maternal Health Outcomes of Digital Health Interventions in Low- and Middle-Income Countries in Southeast Asia: Scoping Review
Source: Asian Pac Isl Nurs J. 2026 Nov 3;10:e92476. doi: 10.2196/92476 (PMC13730517; doi:10.2196/92476)
Supplement: Supplementary file 1 [file apinj-v10-e92476-s001.docx]

**Multimedia Appendix 1. Full Search Strategies**

**Search Sources, Rationale, and Timeframe**

PubMed, CINAHL, and Scopus were prespecified to provide complementary coverage of biomedical, nursing and allied health, and multidisciplinary literature. Google Scholar and reference-list screening were used to supplement the database searches. Web of Science, Embase, Global Health, individual national databases, and dedicated grey literature sources were not searched to maintain a feasible review scope.

PubMed, CINAHL, and Scopus were searched on May 26, 2025, and Google Scholar was searched on June 28, 2025. The searches were limited to English-language publications from January 2005 through May 2025.

A supplementary search of the World Health Organization Global Index Medicus was conducted on August 12, 2026. The interface permitted publication dates to be limited by years only; therefore, the search was limited to records published from 2005 through 2025. The review’s prespecified cutoff of May 31, 2025, was applied during eligibility screening.

**Protocol Registration and Subsequent Search Update**

The review protocol was registered with the Open Science Framework before title and abstract screening began. The supplementary IMSEAR search was added after protocol registration in response to peer review to strengthen regional coverage. The same eligibility criteria and screening procedures were applied, and no additional eligible studies were identified.

**Source-Specific Search Strategies**

**PubMed**

**Date searched: May 26, 2025**

("Maternal Health"[MeSH] OR "Pregnancy"[MeSH]

OR "maternal health" OR pregnancy OR "antenatal care" OR "postnatal care"

OR "maternity care" OR childbirth OR postpartum OR antepartum)

AND

("Telemedicine"[MeSH] OR "Cell Phones"[MeSH]

OR "digital health" OR mHealth OR "mobile health" OR eHealth

OR "mobile application*" OR SMS OR "text messaging"

OR "interactive voice response")

AND

("Southeastern Asia"[MeSH] OR "Southeast Asia"

OR Cambodia OR Indonesia OR "Lao PDR" OR Malaysia OR Myanmar OR Philippines OR Thailand OR Timor-Leste OR Vietnam)

**Limits applied:**

- Language: English
- Publication timeframe: January 2005 through May 2025

**Records retrieved and imported into Covidence**: 455

**CINAHL**

**Date searched: May 26, 2025**

The CINAHL search combined subject headings (MH) and keywords in three concept blocks.

Block 1: Maternal Health

(MH "Maternal Health+") OR (MH "Pregnancy+") OR "maternal health" OR pregnancy OR "antenatal care" OR "postnatal care" OR "maternity care" OR childbirth OR postpartum OR antepartum

Block 2: Digital Health

(MH "Telemedicine+") OR (MH "Mobile Applications+") OR "digital health" OR mHealth OR "mobile health" OR eHealth OR SMS OR "text messaging" OR "interactive voice response"

Block 3: Southeast Asia

(MH "Southeastern Asia+") OR "Southeast Asia" OR Cambodia OR Indonesia OR "Lao PDR" OR Malaysia OR Myanmar OR Philippines OR Thailand OR Timor-Leste OR Vietnam

Final combination: Block 1 AND Block 2 AND Block 3

**Limits applied:**

- Language: English
- Publication timeframe: January 2005 through May 2025

**Records retrieved and imported into Covidence**: 110

**Scopus**

**Date searched: May 26, 2025**

The Scopus search was conducted using TITLE-ABS-KEY fields.

TITLE-ABS-KEY ("maternal health" OR pregnancy OR "antenatal care" OR "postnatal care" OR "maternity care" OR childbirth OR postpartum OR antepartum)

AND

TITLE-ABS-KEY ("digital health" OR mHealth OR "mobile health" OR telemedicine

OR eHealth OR "mobile application*" OR SMS OR "text messaging" OR "interactive voice response")

AND

TITLE-ABS-KEY ("Southeast Asia" OR Cambodia OR Indonesia OR "Lao PDR" OR Malaysia OR Myanmar OR Philippines OR Thailand OR Timor-Leste OR Vietnam)

**Limits applied:**

- Language: English
- Publication timeframe: January 2005 through May 2025

**Records retrieved and imported into Covidence**: 300

**Google Scholar**

**Date searched: June 28, 2025**

Google Scholar was searched using the following keyword query:

"maternal health" AND "digital health" AND ("Southeast Asia" OR Indonesia OR Philippines OR Vietnam OR Cambodia OR Myanmar OR "Lao PDR" OR Thailand OR Malaysia OR Timor-Leste) AND (intervention OR implementation OR outcomes)

Search details:

- Language: English
- Publication timeframe: January 2005 through May 2025
- Number of searches: 1
- Results returned: approximately 4,080
- Result order: Google Scholar’s default relevance ranking
- Records retained: first 200 results

Following published guidance, the first 200 relevance-ranked records were retained [1] and underwent the same deduplication and eligibility-screening procedures.

**World Health Organization Global Index Medicus**

**Date searched: August 12, 2026**

The World Health Organization Global Index Medicus was searched using the Index Medicus for the South-East Asia Region (IMSEAR).

**Search field:** Title, abstract, and subject

**Search term**: Maternal Health

**Filters applied**

- Regional index: IMSEAR (South-East Asia)
- Main subjects
  - Maternal Health Services
  - Maternal Mortality
  - Prenatal Care
  - Maternal and Child Health
- Language: English
- Publication year: 2005–2025

**Records retrieved and imported into Covidence**: 68

**Additional Search Procedures**

- Search strategies were adapted to the syntax and indexing requirements of each source.
- Records imported into Covidence were combined and deduplicated before screening.
- Reference lists of relevant reviews were screened to identify additional eligible studies.
- Final study selection and screening results are reported in the PRISMA-ScR flow diagram in the main manuscript.

**Reference**

1. Haddaway NR, Collins AM, Coughlin D, Kirk S. The role of Google Scholar in evidence reviews and its applicability to grey literature searching. *PLoS One*. 2015;10(9):e0138237. doi:10.1371/journal.pone.0138237
